# TAZ facilitates breast tumor growth by promoting an immune‐suppressive tumor microenvironment

**DOI:** 10.1002/1878-0261.13525

**Published:** 2023-10-16

**Authors:** Anat Gershoni, Ori Hassin, Nishanth Belugali Nataraj, Sivan Baruch, Adi Avioz‐Seligman, Anna Chiara Pirona, Liat Fellus‐Alyagor, Tomer Meir Salame, Saptaparna Mukherjee, Giuseppe Mallel, Yosef Yarden, Yael Aylon, Moshe Oren

**Affiliations:** ^1^ Department of Molecular Cell Biology Weizmann Institute of Science Rehovot Israel; ^2^ Department of Immunology and Regenerative Biology Weizmann Institute of Science Rehovot Israel; ^3^ Department of Veterinary Resources Weizmann Institute of Science Rehovot Israel; ^4^ Flow Cytometry Unit, Department of Life Sciences Core Facilities Weizmann Institute of Science Rehovot Israel

**Keywords:** breast cancer, Hippo pathway, TAZ, Tregs

## Abstract

The core Hippo pathway module consists of a tumour‐suppressive kinase cascade that inhibits the transcriptional coactivators Yes‐associated protein (YAP) and WW domain‐containing transcription regulator protein 1 (WWTR1; also known as TAZ). When the Hippo pathway is downregulated, as often occurs in breast cancer, YAP/TAZ activity is induced. To elaborate the roles of TAZ in triple‐negative breast cancer (TNBC), we depleted *Taz* in murine TNBC 4T1 cells, using either CRISPR/Cas9 or small hairpin RNA (shRNA). TAZ‐depleted cells and their controls, harbouring wild‐type levels of TAZ, were orthotopically injected into the mammary fat pads of syngeneic BALB/c female mice, and mice were monitored for tumour growth. TAZ depletion resulted in smaller tumours compared to the tumours generated by control cells, in line with the notion that TAZ functions as an oncogene in breast cancer. Tumours, as well as their corresponding *in vitro* cultured cells, were then subjected to gene expression profiling by RNA sequencing (RNA‐seq). Interestingly, pathway analysis of the RNA‐seq data indicated a TAZ‐dependent enrichment of ‘Inflammatory Response’, a pathway correlated with TAZ expression levels also in human breast cancer tumours. Specifically, the RNA‐seq analysis predicted a significant depletion of regulatory T cells (Tregs) in TAZ‐deficient tumours, which was experimentally validated by the staining of tumour sections and by quantitative cytometry by time of flight (CyTOF). Strikingly, the differences in tumour size were completely abolished in immune‐deficient mice, demonstrating that the immune‐modulatory capacity of TAZ is critical for its oncogenic activity in this setting. Cytokine array analysis of conditioned medium from cultured cells revealed that TAZ increased the abundance of a small group of cytokines, including plasminogen activator inhibitor 1 (Serpin E1; also known as PAI‐1), CCN family member 4 (CCN4; also known as WISP‐1) and interleukin‐23 (IL‐23), suggesting a potential mechanistic explanation for its *in vivo* immunomodulatory effect. Together, our results imply that TAZ functions in a non‐cell‐autonomous manner to modify the tumour immune microenvironment and dampen the anti‐tumour immune response, thereby facilitating tumour growth.

## Graphical abstract

High expression of the *TAZ* gene in triple‐negative breast cancer patients are associated with poor patient survival. We now report that, in such cancers, TAZ enforces an immunosuppressive tumour microenvironment, characterized by elevated presence of regulatory T cells (Tregs). Depletion of *TAZ* from the cancer cells activates an anti‐tumour adaptive immune response, leading to cancer cell killing and tumour shrinkage.

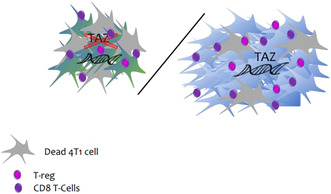

**Abbreviations**
C‐CAS3: cleaved caspase 3
CCN4: Cellular Communication Network Factor 4, CCN family member 4
CTGF: connective tissue growth factor
CyTOF: cytometry by time of flight
DC: dendritic cells
DECODR: DEconvolution of COmplex DNA Repair
FDR: false discovery rate
FOXP3: forkhead box P3
GSEA: Gene Set Enrichment Analysis
HTAZq: highest quartile TAZ expression
IACUC: Institutional Animal Care and Use Committee
IFNγ: interferon gamma
Il‐23: interleukin 23
KO: knock out
LTAZq: lowest quartile TAZ expression
MDSC: myeloid derived suppressor cell
NS: not significant
PI: propidium iodide
RFS: relapse‐free survival
RNA‐seq: RNA sequencing
RT: room temperature
sgRNA: single guide RNAs
ShC: RNA short hairpin control
ShRNA: short hairpin RNA
TCGA: The Cancer Genome Atlas
TME: tumour microenvironment
TNBC: triple negative breast cancer
Treg: regulatory T cell
WT: wild type
YAP: Yes‐associated protein

## 1 Introduction

The tumour microenvironment (TME) is a complex cellular ecosystem orchestrated by cancer cells to support or suppress tumour growth. Different types of tumour‐infiltrating immune cells differentially modulate cancer progression, some driving antitumour immunity while others actually suppress antitumour immunity [1]. A variety of cells, including, but not limited to, macrophages, dendritic cells (DC), neutrophils, B cells, T cells and cancer‐associated fibroblasts, are recruited to the TME [1]. Cytotoxic CD8^+^ T cells have a pivotal role in tumour cell death and clearance. CD8^+^ T cell functionality is dependent on priming and activation; both processes occur in the TME to produce an efficient antitumoral immune response [2]. However, more often than not, T cells in the TME are poorly primed, resulting in a dysfunctional differentiated state termed “exhausted”, thus failing to control tumour growth and subsequently facilitating tumour progression [2]. regulatory T cells (Tregs) suppress the function of immune effector cells, such as CD8^+^ T cells, by a variety of mechanisms, making them key factors in tumour immune surveillance. Specifically, Tregs can cause CD8^+^ T cell exhaustion via secretion of cytokines [3]. Inducible cytokines play a major role in intercellular communication within the immune system, as well as in the crosstalk between immune cells and cancer cells within the TME [4]. Many studies have collectively shown that cytokines and their receptors are often deregulated under pathological conditions, including in cancer [5]. In particular, cytokines present in the TME may promote a tumour supportive microenvironment via suppression of antitumour immunity, accelerating tumour growth and progression [4].

The Hippo pathway is a conserved serine/threonine kinase cascade that controls organ size by regulating its two main downstream effectors, YAP and TAZ. Hippo‐mediated phosphorylation of YAP or TAZ results in their cytoplasmic retention and proteasomal degradation [6, 7, 8]. Under conditions in which the Hippo kinases are inactive, YAP and TAZ translocate to the nucleus and, together with transcription factors such as the TEAD family members, induce the transcription of genes related to migration and growth [8, 9, 10, 11, 12]. Hippo pathway deregulation has been associated with various types of malignancies, including lung, liver, ovarian and breast cancer [6, 13]. Compared to YAP, TAZ is relatively understudied and its role in tumour development has been less extensively characterized.

Breast cancer is the most prominent cancer type for women, accounting for about 30% of all newly diagnosed cases every year. Additionally, it is the second leading cause of cancer‐related death among women [14]. Enhanced TAZ transcriptional activity in breast cancer is correlated with high histological grade, enrichment of stem cell signature, high tendency to develop metastases and poor survival [15, 16]. Importantly, TAZ has been shown to regulate tumour‐immune crosstalk in the TME, mainly via its influence on T cells and macrophages [17]. Additionally, it was recently reported that expression of TAZ correlates strongly with the expression of proinflammatory cytokines [18, 19, 20, 21]. Collectively, these observations suggest that TAZ may play a critical role in the interaction between cancer cells and immune cells in the TME.

In the present study, we set out to explore the role of TAZ in breast cancer development. We report that TAZ regulates a distinct transcriptional program, favouring evasion of cancer cells from immune surveillance and resulting in accelerated tumour growth. Specifically, TAZ drives the production of a small set of cytokines, which possibly promote a tumour supportive TME. Overall, our findings support the conclusion that, in addition to its well‐documented cell‐autonomous oncogenic activities, TAZ can also contribute to tumour progression via cell non‐autonomous mechanisms.

## 2 Materials and methods

### 2.1 Generation of TAZ‐KO and shTAZ mouse breast cancer cell clones

4T1 cells (RRID:CVCL_0125) were purchased from ATCC. All experiments were performed with mycoplasma‐free cells. To generate knockout clones, a CRISPR/Cas9 two plasmid system was used [22]. Initially, 4T1 cells, which possess a tetraploid genome [23], were infected with a recombinant lentivirus harbouring the gene for Cas9. These cells were selected with 5 μg·mL^−1^ blasticidin and then seeded in a 96‐well culture plate to obtain single clones of 4T1‐Cas9 cells. A single cell clone with prominent expression of Cas9 was selected for subsequent infections with a lentivirus expressing single guide RNAs (sgRNAs) targeting sites within the mouse *Taz* (*Wwtr1*) gene. These cells were re‐selected with 1 μg·mL^−1^ puromycin, followed by single cell clone selection. Altogether, two independent *Taz* knockout (KO) clones, with the same sgRNA, were obtained. As wild type (WT) controls, we used single cell clones of 4T1‐Cas9 cells not infected with sgRNA (Fig. S1a). Successful editing of all four *Taz* alleles was confirmed by DNA sequencing and analysis using DEconvolution of COmplex DNA Repair (DECODR) [24].

To obtain inducible knockdown of *Taz*, 4T1 cells were stably transduced with lentiviruses driving doxycycline‐inducible expression of TAZ‐targeting shRNA (shTAZ), or of scrambled shRNA as control (shC); sequences are listed in Table S1. Cells were selected with 1 μg·mL^−1^ puromycin. Expression of the shRNA was induced *in vitro* by 48 h treatment with 6 mm doxycycline (Sigma‐Aldrich, #24390‐14‐5, St. Louis, MO, USA), and *in vivo* by feeding the mice with chow containing doxycycline (TD.01306, 625 mg·kg^−1^).

4T1 cells were obtained directly from ATCC and were immediately expanded with minimal passaging, frozen in aliquots and stored in liquid nitrogen. For subsequent experiments, frozen cells were thawed and grown with minimal *in vitro* passaging.

### 2.2 Western blot analysis

Western blot analysis was performed as described [25], using the following antibodies: GAPDH (Cell Signaling, Catalog #2118), YAP/TAZ (Cell Signaling, Catalog #8418), TAZ (Cell Signaling, Catalog #4883). Conjugated anti‐rabbit secondary antibodies were from Jackson ImmunoResearch. Imaging and quantification were performed using a ChemiDoc MP Imager with image lab 4.1 software (Bio‐Rad, CA, USA).

### 2.3 Immunohistochemistry

Following fixation, samples were stained with haematoxylin and eosin (H&E, Sigma, Catalog #HHS332 and Catalog #HT110332), anti‐CD3 (Abcam, MA, USA, Catalog #ab16669), anti‐CD8a (eBioscience, CA, USA, Catalog #14‐0808‐82), anti‐FOXP3 (eBioscience, Catalog #14‐5773‐82), anti‐Ki67 (Abcam, Catalog #ab16667) and anti‐Cleaved Caspase‐3 (Cell Signaling Technology, Catalog #9661L). Slides were imaged using a Leica Mi8 microscope equipped with a motorized stage and a Leica DFC365 FX camera. Single 10× magnification images were tiled to obtain a full scan of the tumour section. Staining intensity was quantified using imagej software [26].

### 2.4 RNA isolation and RT‐qPCR analysis

RNA was isolated using a NucleoSpin kit (Macherey Nagel, Duren, Germany, 740955.50). 1–2 μg of each RNA sample was reverse transcribed using LunaScript (LunaScript ^®^ RT SuperMix Kit, NEB‐E3010L). Reverse transcriptase qPCR (RT‐qPCR) was performed using SYBR Green PCR Supermix (Thermo Fisher Scientific, MA, USA, #4385614) with a StepOne real‐time PCR instrument (Applied Biosystems, MA, USA). For each gene, values for the standard curve were measured and the relative quantity was normalized to *HPRT* or *GAPDH* mRNA. Primers are listed in Table S2.

### 2.5 RNA library preparation and sequencing

A bulk adaptation of the MARS‐Seq protocol [27] was used to generate RNA‐seq libraries. Briefly, 30 ng of input RNA from each sample was barcoded during reverse transcription and pooled. Following Agencourt AMPure XP beads cleanup (Beckman Coulter, CA, USA), the pooled samples underwent second strand synthesis and were linearly amplified by T7 *in vitro* transcription. The resulting RNA was fragmented and converted into a sequencing‐ready library by tagging the samples with Illumina sequences during ligation, RT and PCR. Libraries were quantified by Qubit (Thermo Fisher Scientific) and TapeStation (Agilent, CA, USA). Sequencing was done with a Nextseq 75 cycles high output kit (Illumina, CA, USA).

RNA‐sequencing (RNA‐seq) analysis was performed using the UTAP transcriptome analysis pipeline [28]. Reads were trimmed to remove adapters and low‐quality bases using cutadapt [29] and mapped to genome GRCm38 (Gencode, UCSC) using star v2.4.2a [30]. Counts normalization and differential expression detection were performed with DESeq2 [31]. For *in vitro* RNA‐seq, batch correction was done using the Surrogate Variable Analysis (sva; 3.26.0) r package. Gene Set Enrichment Analysis was performed using the gsea [32] preranked tool. CIBERSORT was used to deconvolute bulk RNA data from tumour samples, predicting the relative abundance of immune cell components that comprise the TME [33].

### 2.6 Animals

The mouse strains used in this study were BALB/c inbred mice (BALB/cOlaHsd), NSG mice (NOD.CB17‐Prkdcscid/NCrHsd), and nude mice (Hsd:Athymic Nude‐Foxn1nu/Foxn1^+^). Mice were purchased from ENVIGO, Jerusalem. All experiments were conducted with 8‐week‐old female mice.

All mouse experiments were approved by the Institutional Animal Care and Use Committee (IACUC) of the Weizmann Institute (application no: 01260120‐2). Housing and handling conditions were as follows: the temperature in the animal rooms was 21 °C with 50% humidity, and animals were maintained under alternating cycles of 12 h of light and 12 h of darkness. Sentinels were placed in all carts to control for good health conditions in the unit. Numbers of mice in each experimental group are listed in Table S3.

To knock down TAZ in the tumours of BALB/c or NSG mice, animals were pre‐fed with chow diet containing doxycycline (TD.01306, 625 mg·kg^−1^) for 3 days prior to injection, and maintained on the same diet for the entire duration of the experiments.

### 2.7 Human data analysis

TCGA breast invasive carcinoma gene expression clinical data [IlluminaHiSeq log (normalized counts +1)] were downloaded from the Xena cancer genome browser (http://xena.ucsc.edu). Triple negative breast cancer (TNBC) tumours were defined as oestrogen, progesterone and HER negative. TNBC samples were analysed by comparing the 25% of tumours expressing the highest levels of TAZ (HTAZq) vs. the 25% of tumours expressing the lowest levels (LTAZq). Differences between groups of tumours were examined by *t*‐test. Several gene signatures adapted from the literature were then compared between the HTAZq and LTAZq groups. In Xena genomic signatures, expressed as a weighted sum of genes, once a signature was entered, the value for each gene name for each sample was substituted with an algebraic value corresponding to expression. Correlation analysis was done using Xena Pearson correlation analysis.

Survival plots were generated with the Kaplan–Meier plotter tool [34] using the following probes: TAZ 202132_at, WISP1 211312_s_at, SERPINE1 202627_s_at, IL23a 220054_at. Tumours were divided according to expression (median cutoff). When comparing groups of genes as a signature, z‐scores of the expression were calculated per each gene, and the average of these *z*‐scores per sample was used for comparisons.

### 2.8 Cytokine array

Cytokine array analysis was performed with a proteome profiler array (Mouse XL Cytokine Array Kit, R&D Systems, MN, USA, #ARY028). Cell supernatants were collected 48 h after seeding. Quantification of the blot membrane was performed with imagej software, normalizing the dot intensity compared to control dot intensity minus the background intensity. Each cytokine was represented in duplicate, and the average normalized value was used for quantification.

### 2.9 Cytometry by time of flight (CyTOF)

4T1 tumour samples were dispersed into single‐cell suspensions prior to analysis. Mechanical and enzymatic dissociation was performed using the tough tumour dissociation protocol on a GentleMACS Dissociator and the Multi Tissue Dissociation Kit 2 (37C_M_TDK_2, Miltenyi, Bergisch Gladbach, Germany) according to the manufacturer's instructions. After tissue dissociation, single‐cell suspensions were filtered through 70 μm meshes to remove non‐dissociated clumps. Red blood cell lysis solution (#130‐094‐183, Miltenyi) was used to remove red blood cells; the solution was applied to the filters, incubated with the single‐cell suspensions and then washed, according to manufacturer's instructions.

The number of viable cells in the single‐cell suspensions was assessed using Trypan Blue. Immune cell enrichment was then performed by depletion of epithelial tumour cells, using CD326 (EpCAM) MicroBeads (#130‐105‐958, Milteny). Briefly, CD326 magnetic beads in phosphate‐buffered saline (PBS; pH 7.2, 0.5% BSA, 2 mm EDTA) were incubated with the cell suspensions for 15 min at 4 °C. Labelled and non‐labelled cells were collected using magnetic separating LS columns (#130‐042‐401, Milteny) and QuadroMACS Separator (#130‐091‐051, Milteny) according to the manufacturer's instructions. RNA was extracted and reverse‐transcribed from the EpCAM^+^ epithelial tumour cells, and TAZ expression and transcriptional activity were evaluated by RT‐qPCR of TAZ and CTGF, respectively.

EpCAM‐negative cells were washed with Maxpar PBS (Fluidigm, CA, USA, #201058) and transferred to a 96‐Well Conical Bottom Plate (#249952, Thermo Fisher Scientific) to reduce cell loss. Two washes were performed with DMEM +10% FBS and with Maxpar Cell Staining Buffer (Fluidigm # 201068). To prevent non‐specific binding, Fc receptors were blocked before staining using anti‐CD16/32 antibody (#101302, BioLegend), according to the manufacturer's instructions. Next, about 1 × 10^6^ cells per sample were incubated with the extracellular antibodies cocktail for 45 min on ice. Cells were washed with Maxpar Cell Staining Buffer (Fluidigm #201068) and with Maxpar PBS (Fluidigm #201058), labelled with 1.25 μm Cell‐ID—Cisplatin (Fluidigm #201064) for 1 min to stain for dead cells, and counted. Then, cells were fixed with fresh 1.6% paraformaldehyde (from a stock of 16% paraformaldehyde, #15710, Electron Microscopy Sciences, in PBS) and incubated at 4 °C overnight.

Next, the cells were fixed and permeabilized with the Maxpar Nuclear Antigen Staining Buffer Set (Fluidigm #201063), followed by counting. Cell barcoding was then performed with the Cell‐ID 20‐Plex Pd Barcoding Kit (Fluidigm #201060): cells were incubated with 1–2 μL barcode in Maxpar Nuclear permeabilization buffer (from the Antigen Staining Buffer Set) for 1 h at room temperature (RT). The cells were then washed twice with the Maxpar Nuclear permeabilization buffer and the barcoded samples were combined. Next, cells were washed again with Maxpar Nuclear permeabilization buffer, and 10% horse serum in Maxpar Nuclear permeabilization buffer and 10% FBS in PBS were added to reduce non‐specific background. Then, the cells were incubated with the intracellular antibodies cocktail for 45 min at RT, washed twice with Maxpar Cell Staining Buffer and fixed with fresh 4% paraformaldehyde (from 16% paraformaldehyde, #15710, Electron Microscopy Sciences, in PBS) at 4 °C for 3 days. The paraformaldehyde solution was then supplemented with Cell‐ID Intercalator‐Ir (Fluidigm #201192A) at a final concentration of 125 nm and incubated for 45 min at RT to label DNA. The cells were then washed twice with Maxpar Cell Staining Buffer followed by Maxpar Cell Acquisition Solution (Fluidigm # 201240), at a concentration of about 300 K cells·mL^−1^, and filtered through a 35 μm mesh. Data was acquired via a Fluidigm CyTOF Helios platform. Metal conjugated antibodies were purchased from Fluidigm or conjugated in‐house using the MIBItag Conjugation Kit (IONPATH). Analysis of the CyTOF data was performed using flowjo. Cytobank Gating editor was used to separate positive populations.

### 2.10 Statistics

All value points of all line and bar graphs are mean ± SEM unless noted otherwise. The significance of all averages presented in the bar or line graphs was determined with *t*‐test. *P*‐values are denoted as follows: *P* < 0.05 (*), *P* < 0.01 (**), *P* < 0.005 (***), not significant (NS).

## 3 Results

### 3.1 Diminished expression of TAZ decreases tumour size

**Fig. 1:**
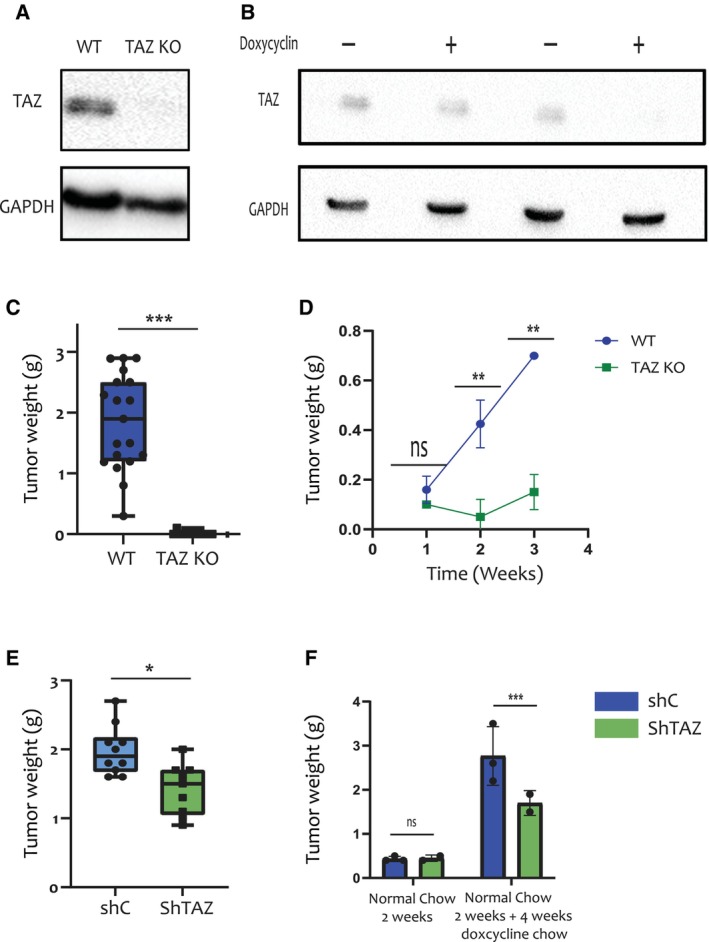
TAZ is required for optimal growth of 4T1‐derived mammary tumours. (A) Western blot analysis of TAZ (Cell Signaling, Catalog #4883) and GAPDH proteins in representative WT and TAZ‐KO 4T1 single cell clones (*n* = 3). (B) Western blot analysis of TAZ and GAPDH proteins in control shRNA (shC) and shTAZ 4T1 cell pools (*n* = 3), harvested after 48 h with or without doxycycline (6 μm). (C) WT and TAZ‐KO 4T1 cells (0.25 × 10^6^) were orthotopically injected into the mammary fat pads of BALB/c mice. Tumours were excised and weighed 6 weeks later. TAZ‐KO tumours were undetectable. Cumulative data from 17 mice injected with either of 3 WT clones and 12 mice injected with either of 2 TAZ‐KO clones. Error bars indicate mean ± SEM from all repeats. ***P < 0.001, Unpaired *T*‐test. (D) WT and TAZ‐KO 4T1 cells (1 clone of each, 0.25 × 10^6^) were injected as in (C). Tumours were harvested at the indicated time points and weighed. Numbers of mice were: 1 week, 5 WT and 2 TAZ‐KO mice; 2 weeks: 4 WT and 2 TAZ‐KO mice; 3 weeks: 3 WT and 2 TAZ‐KO mice. Error bars indicate mean ± SEM from all repeats. **P < 0.01, Unpaired *T*‐test. (E) 4T1 cells harbouring inducible shRNA, either shC or shTAZ, were treated *in vitro* for 48 h with 6 μm doxycycline, and 0.25 × 10^6^ cells were orthotopically injected into the mammary fat pads of BALB/c mice pre‐fed for 2 weeks with doxycycline chow (625 mg·kg^−1^). Mice were maintained on doxycycline chow for 6 weeks, after which tumours were excised and weighed. Data was compiled from 9 shTAZ and 10 shC mice. Error bars indicate mean ± SEM from all repeats. *P < 0.05, Unpaired *T*‐test. (F) shC and shTAZ 4T1 cells were orthotopically injected (*n* = 3 for ShC group and *n* = 2 for ShTAZ group) into BALB/c mice (0.25 × 10^6^ cells per mouse). Mice were maintained on normal chow for 2 weeks, and then either sacrificed for tumour analysis (left) or switched to doxycycline chow for 4 more weeks (right; *P*‐value = 0.004). Error bars indicate mean ± SEM from all repeats. ***P < 0.001, Unpaired *T*‐test. KO, knockout; ShC, scrambled shRNA; ShTAZ, *TAZ*‐targeting shRNA; WT, wild type.

To explore biological mechanisms that might account for the association of high TAZ expression with poor survival of breast cancer patients, we depleted the *Taz* gene from mouse 4T1 breast cancer cells using CRISPR/Cas9, to generate TAZ knockout (TAZ‐KO) cells. The sgRNA sequence and the targeted location in the *Taz* (*Wwtr1*) coding region are shown in Fig. S1a. Genomic DNA sequencing confirmed effective targeting of all four *Taz* alleles of the tetraploid 4T1 cells (Fig. S1b). In agreement, RT‐qPCR analysis showed a significant decrease in *Taz* mRNA, compared to WT control clones (Fig. S1c), presumably owing to nonsense‐mediated RNA decay. As expected, the TAZ‐KO cells displayed complete loss of TAZ protein (Fig. 1A). In parallel, we generated 4T1 cells with inducible knockdown of TAZ (shTAZ), which were maintained as a cell pool. Treatment of the shTAZ cells *in vitro* for 48 h with 6 μm doxycycline resulted in a significant decrease in *Taz* mRNA (Fig. S1d) and a marked reduction in TAZ protein (Fig. 1B), compared to shRNA control (shC) cells.

Next, we injected TAZ‐KO and shTAZ cells, and their corresponding control cells, into the mammary fat pads of female BALB/c mice. Strikingly, by 6 weeks after injection, no tumours could be observed in the mice injected with the TAZ‐KO cells, in contrast to the large tumours that developed in mice injected with their wild type (WT) counterparts (Fig. 1C). Excision of tumours at earlier time points revealed a failure of tumours to grow in the TAZ‐KO‐injected mice as compared to the WT‐injected mice, which was already evident as early as 2 weeks post injection (Fig. 1D). Together, these observations suggested that depletion of TAZ severely restricts tumour growth at initial stages and presumably leads to eventual complete elimination of these tumours. Similarly, orthotopic injection of shTAZ cells into mice that were continuously fed with doxycycline chow resulted in decreased tumour size compared to control shRNA (shC) (Fig. 1E). To obtain further temporal resolution of this process, BALB/c mice injected with either shTAZ or shC cells were fed with normal chow for 2 weeks, at which time half of the mice were sacrificed. As seen in Fig. 1F, under those conditions, when TAZ shRNA expression was not induced, shTAZ and shC tumours were similar in size. The rest of the mice were then switched to doxycycline‐containing chow for an additional 4 weeks, to silence TAZ expression in the shTAZ tumours. Remarkably, this resulted in a significant growth retardation of the shTAZ tumours relative to their shC controls (Fig. 1F), implying that TAZ is required not only for tumour establishment but also for optimal continued growth in a syngeneic host.

### 3.2 TAZ‐dependent tumour growth is associated with diminished apoptosis

**Fig. 2:**
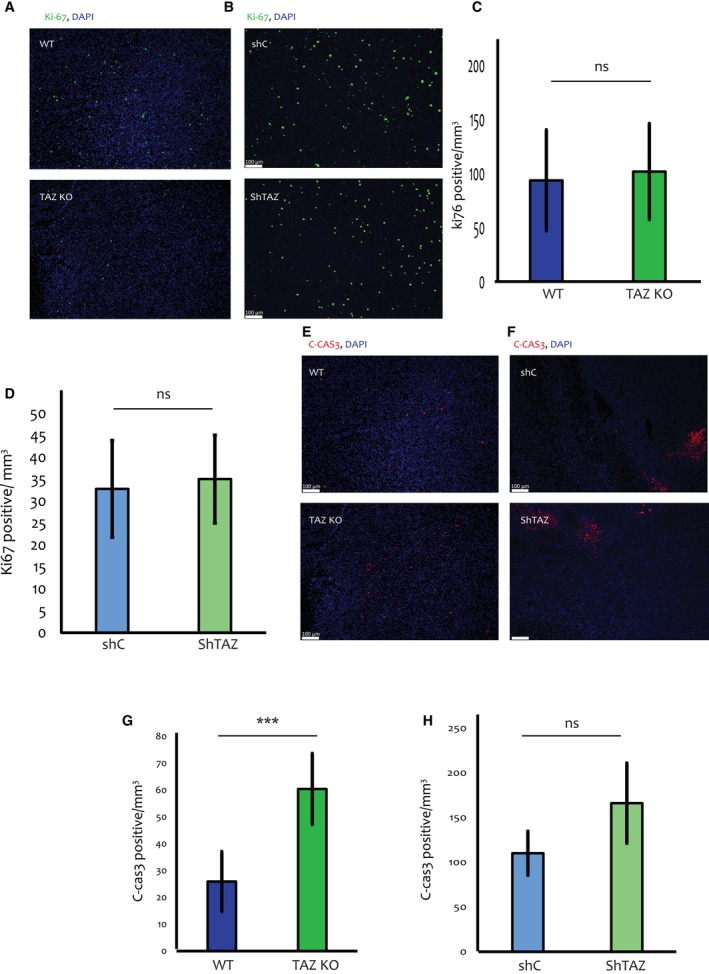
TAZ depletion results in increased apoptosis in 4T1 tumours. (A) Representative images (10× magnification) of Ki67 (green) and DAPI (blue) staining of sections from either WT and TAZ‐KO tumours (*n* = 4 per group) harvested 2 weeks post injection into BALB/c mice. Scale bar = 100 μm. (B) Representative images (10× magnification) of Ki67 and DAPI staining of shC and shTAZ tumours (*n* = 5 per group) induced as in Fig. 1E. (C) Quantification of Ki67‐positive cells in WT and TAZ‐KO tumours, compiled from entire slide scans of 4 WT and 4 TAZ‐KO tumours excised 2 weeks post injection, as well as 2 WT and 2 TAZ‐KO tumours excised 3 weeks post injection. Ki67‐positive cells were counted using the imagej MOMENT threshold and normalized to the measured area using imagej polygon selection. Error bars indicate mean ± SEM from all repeats. *P* > 0.05, Unpaired *T*‐test. (D) Quantification of Ki67‐positive cells in shC and shTAZ tumours (5 tumours each), harvested after 6 weeks of feeding with doxycycline chow (625 mg·kg^−1^). Analysis was performed as in (C). Error bars indicate mean ± SEM from all repeats. *P* > 0.05, Unpaired *T*‐test. (E) Images of C‐Cas3 (red) and DAPI (blue) staining of sections [tissue sections similar to (A)] from representative WT and TAZ‐KO tumours (*n* = 4 per group), harvested 2 weeks post injection into BALB/c mice. Scale bar = 100 μm. Tissue section are similar to those in (A). (F) Images of C‐Cas3 and DAPI staining of sections from representative shC and shTAZ tumours (*n* = 5 per group) harvested 6 weeks post injection. Scale bar = 100 μm. (G) Quantification of C‐Cas3 staining in WT vs. TAZ KO tumours. Values were compiled from entire slide scans of 4 WT and 4 TAZ‐KO tumours excised 2 weeks post injection, as well as 2 WT and 2 TAZ‐KO tumours excised 3 weeks post injection. C‐Cas3‐positive cells were counted using imagej MOMENT threshold and normalized to the measured area using imagej polygon selection. Error bars indicate mean ± SEM from all repeats. ***P < 0.001, Unpaired *T*‐test. (H) Quantification of C‐Cas3 staining in shC and shTAZ tumours, done as in (G). Data compiled from 5 shC and 5 shTAZ tumours, induced as in (D) and harvested after 6 weeks. Error bars indicate mean ± SEM from all repeats. *P* > 0.05, Unpaired *T*‐test. C‐Cas3, cleaved caspase 3; KO, knockout; ShC, scrambled shRNA; ShTAZ, *TAZ*‐targeting shRNA; WT, wild type.

To elucidate the biological processes that may contribute to TAZ‐dependent tumour growth, we first tested whether TAZ drives increased proliferation of the cancer cells *in vivo*. However, we did not observe significant differences in the abundance of Ki67‐positive cells when comparing WT vs. TAZ‐KO tumours at early time points, when such comparison could still be performed (Fig. 2A,C). Likewise, no differences in Ki67‐positive cell abundance were seen when shC and shTAZ tumours were compared 6 weeks post‐injection (Fig. 2B,D). Thus, it is unlikely that enhanced cell proliferation accounts for the tumour promoting effect of TAZ in this model.

Attenuation of cell death could provide an alternative explanation for differences in tumour size. To test this possibility, we employed cleaved caspase 3 (C‐Cas3) staining to monitor apoptosis. Remarkably, 2 or 3 weeks post‐injection, C‐Cas3‐positive cells were significantly more abundant in the TAZ‐KO tumours than in the WT ones (Fig. 2E,G), consistent with the differences in tumour size. Likewise, a trend towards augmented apoptosis was observed in shTAZ tumours relative to shC tumours, in mice continuously fed with doxycycline chow for 6 weeks (Fig. 2F,H). In contrast, analysis of *in vitro* cell death by propidium iodide (PI) staining in shC and shTAZ cells, treated for 48 h with doxycycline, did not reveal any significant differences in cell death (Fig. S1e).

YAP and TAZ share many common downstream targets. It was therefore important to validate that YAP expression was not affected by TAZ depletion. As shown in Fig. S1f, YAP protein levels remained unchanged upon TAZ knockout, ruling out confounding effects of altered YAP expression.

Together, these observations suggest that TAZ may protect the cancer cells against apoptotic death particularly within the context of the tumour microenvironment.

### 3.3 Transcriptional programs driven by TAZ elicit changes in the tumour immune microenvironment

**Fig. 3:**
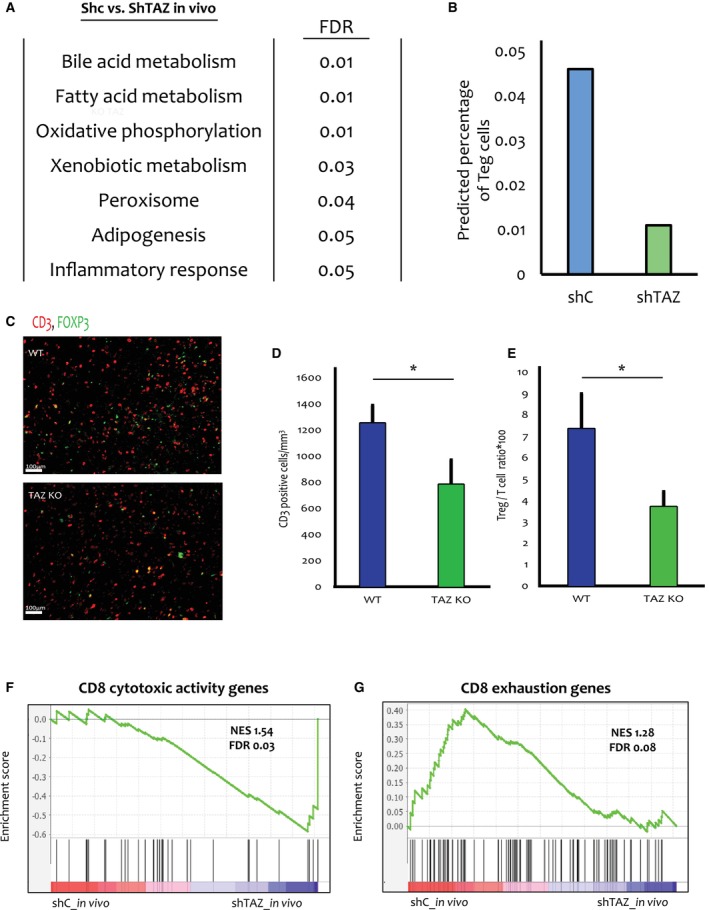
TAZ drives an inflammatory gene signature and an increase in regulatory T cells (Tregs). (A) Tumours induced by *in vitro* treatment of shC or shTAZ cells for 48 h hours with 6 μm doxycycline, followed by mammary fat pad injection and continuous administration of 625 mg·kg^−1^ doxycycline for 6 weeks (4 tumours of each genotype), were subjected to RNA‐seq analysis. Shown are pathways enriched in shC tumours compared to shTAZ tumours (FDR ≤ 0.05), determined by GSEA. (B) The RNA‐seq data in (A) was analysed using the *CIBERSORT* analytical tool [33]. Shown is the predicted percentage of Tregs in the shC vs. shTAZ tumours. (C) Representative images at 10× magnification of WT and TAZ‐KO tumours (*n* = 5 per group) harvested 2 weeks post injection and stained for CD3 (red), FOXP3 (green). Scale bar = 100 μm. (D) Quantification of CD3‐positive cells in WT vs. TAZ‐KO tumours. Values were compiled from entire slide scans of 5 WT and 5 TAZ‐KO tumours harvested 2 weeks post injection. Analysis was performed using the imagej software MOMENT threshold. Error bars indicate mean ± SEM from all repeats. *P < 0.05, Unpaired *T*‐test. (E) Quantification of Tregs (CD3/FOXP3 double positive cells) relative to the total number of CD3^+^ T cells. 15 random tiles were picked from 5 WT and 5 TAZ‐KO tumours. CD3‐positive cells and FOXP3‐positive cells were identified using the imagej MOMENT threshold. Double positive cells were counted manually, and their number was normalized to the total number of CD3‐positive cells. Error bars indicate mean ± SEM from all repeats. *P < 0.05, Unpaired *T*‐test. (F) Gene Set Enrichment Analysis (GSEA) of a CD8 T cell cytotoxic activity gene signature [39] in the *in vivo* shC vs. ShTAZ RNA‐seq data. (G) GSEA of a CD8 T cell exhaustion gene signature [39] in the *in vivo* shC vs. ShTAZ RNA‐seq data. FOXP3, forkhead box P3; GSEA, gene set enrichment analysis; KO, knockout; ShC, scrambled shRNA; ShTAZ, *TAZ*‐targeting shRNA; WT, wild type.

To explore molecular mechanisms that may underlie the biological effects of TAZ, we performed comparative global expression analysis (RNA‐seq) of four shC tumours and four shTAZ tumours, harvested 6 weeks post injection from mice that were continuously fed with doxycycline chow. Additionally, we performed RNA‐seq analysis of shTAZ and shC cells treated *in vitro* with doxycycline for 48 h, as well of TAZ‐KO cells (2 independent clones) and WT control cells (3 independent clones). Gene set enrichment analysis (GSEA) of the RNA‐seq data yielded seven pathways that were enriched with an FDR of 0.05 or lower in the shC tumours relative to the shTAZ tumours (Fig. 3A), suggesting that these pathways are regulated by TAZ *in vivo*. From this GSEA, we generated a gene signature for each enriched pathway, comprising only the differentially expressed genes that contributed to the enrichment of the particular pathway. We then queried which gene signature was also enriched *in vitro* in TAZ‐proficient cells, relative to their corresponding TAZ‐deficient counterparts. Interestingly, this revealed that the gene signature termed “Inflammatory response” was enriched *in vitro* in both shC (albeit with an FDR of 0.07) and WT cells (FDR = 0.01), relative to shTAZ and TAZ‐KO cells, respectively (Fig. S2a,b).

To assess the human relevance of TAZ expression, we analysed TNBC patient data from TCGA. TNBC tumours were binned according to their relative *TAZ* mRNA levels; tumours expressing high *TAZ* mRNA (upper quartile, HTAZq) were compared to tumours expressing low *TAZ* mRNA (bottom quartile, LTAZq). Reassuringly, a published signature of YAP/TAZ transcriptional activity [35] was strongly enriched in the HTAZq tumours compared to the LTAZq tumours, implying that *TAZ* mRNA expression is an acceptable proxy for TAZ transcriptional activity in human TNBC (Fig. S2c). Importantly, the “Inflammatory response” signature was significantly enriched in the HTAZq tumours relative to the LTAZq tumours (Fig. S2d). Altogether, “Inflammatory response” emerged as the only gene signature that was consistently enriched in correlation with TAZ expression throughout all the different systems analysed, suggesting that TAZ expression is intrinsically associated with factors that may increase inflammation in both mouse and human TNBC tumours.

To interrogate the physiological consequences of the TAZ‐associated inflammatory response, we applied CIBERSORT [36] to estimate differences in immune cell composition between shC and shTAZ tumours (Fig. S2e). Notably, this analysis predicted a decrease in Treg abundance upon TAZ silencing (Fig. 3B, Fig. S2e). Similarly, CIBERSORT suggested that Tregs were more abundant in human HTAZq tumours than in LTAZq tumours (Fig. S2e). Furthermore, a published Treg gene signature [37] was also elevated in HTAZq tumours as compared to LTAZq tumours (Fig. S2f). Notably, the Treg signature was positively correlated with the “Inflammatory response” gene signature (*R* = 0.699; Fig. S2g). Hence, TAZ may promote the recruitment of Tregs in both human and mouse TNBC tumours as part of a cancer‐protective inflammatory response.

To assess more directly the impact of TAZ on the tumour immune microenvironment, we subjected tumours from our different mouse models to immunohistochemistry analysis. Interestingly, while no significant differences in T cell abundance were observed in shC vs. shTAZ tumours (Fig. S2h,j), TAZ‐KO tumours analysed 2 weeks post‐injection displayed decreased abundance of infiltrating T cells (CD3^+^) relative to their WT counterparts (Fig. 3C,D). Importantly, analysis of the relative proportion of Tregs (CD3^+^FOXP3^+^) within the entire population of infiltrating T cells (CD3^+^) revealed that Tregs were significantly enriched in the WT and shC tumours relative to the TAZ‐KO and shTAZ tumours, respectively (Fig. 3C,E and Fig. S2i,j). Thus, in both 4T1‐derived models, TAZ appears to enhance Treg infiltration, consistent with the CIBERSORT predictions and the human patient data analysis.

Tregs drive cytotoxic T cell dysfunction [2, 38]. We thus asked whether the increased abundance of Tregs in TAZ‐proficient tumours is associated with augmented cytotoxic T cell dysfunction. Indeed, comparison of shTAZ vs. shC tumours confirmed that a gene set representing the cytotoxic activity of CD8^+^ T cells [39] was enriched upon TAZ depletion (Fig. 3F). Conversely, a gene set indicative of T cell exhaustion [39] tended to be positively associated with retention of TAZ (Fig. 3G). Altogether, these analyses support the conjecture that TAZ promotes recruitment of Tregs to the tumour, thereby suppressing the cytotoxic activity of the infiltrating CD8^+^ T cells. Consequently, TAZ depletion is expected to promote T cell‐mediated cancer cell killing, leading to reduced growth of the shTAZ tumours and eventual elimination of the TAZ‐KO tumours.

### 3.4 TAZ promotes cytokine expression and secretion

**Fig. 4:**
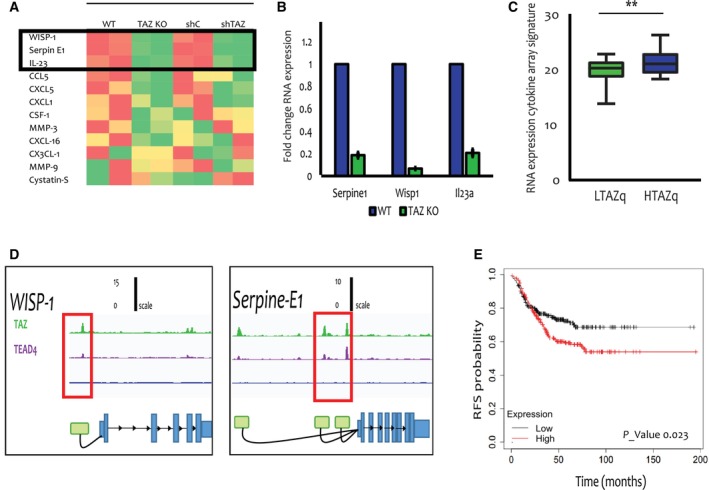
TAZ regulates cytokine expression and secretion. (A) Cytokine array analysis was performed on the conditioned medium of WT vs. TAZ‐KO cells (left columns) or shC vs. shTAZ cells (right columns, after 48 h treatment with 6 μm doxycycline). The heatmap represents the relative score of each informative cytokine (red = high, green = low), based on quantification of the blot with the image lab software; the average of two representative dots for each cytokine was used. The top three cytokines shared between both comparisons are boxed. (B) RT‐qPCR analysis of *Wisp1*, *Serpine1* and *Il23a* mRNA in WT vs. TAZ‐KO cells cultured *in vitro*. Data from three independent repeats. Values were normalized to WT cells, taken as 1.0. Error bars indicate mean ± SEM from all repeats. *P*‐values from left to right: 0.004, 0.003, 0.004. (C) TNBC patient tumours (TCGA BRCA TNBC cohort) were ranked according to *TAZ* mRNA expression; HTAZq and LTAZq represent the top and bottom quartiles (high *TAZ* mRNA and low *TAZ* mRNA), respectively. Expression of the 3‐gene signature comprising the 3 genes in (B) was compared between the HTAZq and LTAZq groups. Error bars indicate mean ± SEM from all repeats. **P < 0.01, Unpaired *T*‐test. (D) Binding of TAZ and TEAD4 to regulatory regions of the *CCN4 (WISP1)* and *SERPINE1* genes in MDA‐MB‐231 cells. ChIP‐seq data [40] (*n* = 2) was reanalysed using the UTAP pipeline [28]. Peak calling was up to 5Kb upstream to the *WISP1* and *SERPINE1* TSS. Green represents TAZ peaks, purple represents TEAD4 peaks and blue represents IgG control peaks. (E) Kaplan–Meier relapse‐free survival (RFS) plot of TNBC patients, comparing low expression (black) vs. high expression (red) of a combined cytokine signature (WISP1 ‐ 211312_s_at, SERPINE1‐ 202627_s_at, IL23a ‐ 220054_at). High and low expressions were defined as being above or below the median, respectively. Log rank test. *CCN4*, CCN family member 4; HTAZq, highest quartile *TAZ* mRNA expression; *Il23*, interleukin 23; KO, knockout; LTAZq, LOWEST quartile *TAZ* mRNA expression; *SerpinE1*, plasminogen activator inhibitor 1; ShC, scrambled shRNA; ShTAZ, *TAZ*‐targeting shRNA; WT, wild type.

Cytokines are pivotal components in the crosstalk between the tumour and the immune system. Specifically, some cytokines can recruit Tregs, while others can drive the exhaustion of CD8^+^ T cells [4], positioning them as potential mediators of the observed TAZ‐regulated alterations in the immune TME. Therefore, we employed cytokine arrays to inquire whether TAZ‐dependent differences in cytokine secretion might instruct differences in the immune TME, Notably, this revealed similar differences in the pattern of cytokine secretion in shTAZ vs. shC cells and in TAZ‐KO vs. WT cells (Fig. S3a,b). In particular three cytokines: Serpin E1 (PAI‐1), IL‐23 and WISP‐1, displayed marked dependence on TAZ expression in both comparisons (Fig. 4A). Moreover, the mRNA levels of these cytokines were downregulated in TAZ‐KO cells (Fig. 4B). A similar trend was observed for *Serpine1* and *Wisp1* mRNA also in doxycycline‐induced shC to shTAZ cells (Fig. S3c). Intriguingly, this was not observed for *Il23a* mRNA, suggesting that the TAZ‐dependent secretion of IL23 from these cells might involve post‐translational mechanisms.

Importantly, the combined expression of these three cytokine genes (cytokine array signature) was augmented in human TNBC tumours expressing high *TAZ* mRNA levels, compared to tumours with low TAZ expression (HTAZq and LTAZq, respectively; Fig. 4C). Concordantly, analysis of published ChIP‐seq data from MDA‐MB‐231 human TNBC cells [40] revealed peaks for both TAZ and TEAD4 at genomic positions corresponding to putative upstream regulatory regions [41] of the *WISP1* and *SERPINE1* genes (Fig. 4D). In contrast, only very weak peaks were observed for the *IL23A* gene (Fig. S3d), suggesting that it may not be a direct TAZ target.

Furthermore, in human breast cancer tumours, the Treg signature was positively correlated (*R* = 0.33) with the cytokine array signature (Fig. S3e). Importantly, high expression of the cytokine array signature was associated with poor survival of TNBC patients (Fig. 4E), consistent with the poor survival of high *TAZ* patients (Fig. S3f).

**Fig. 5:**
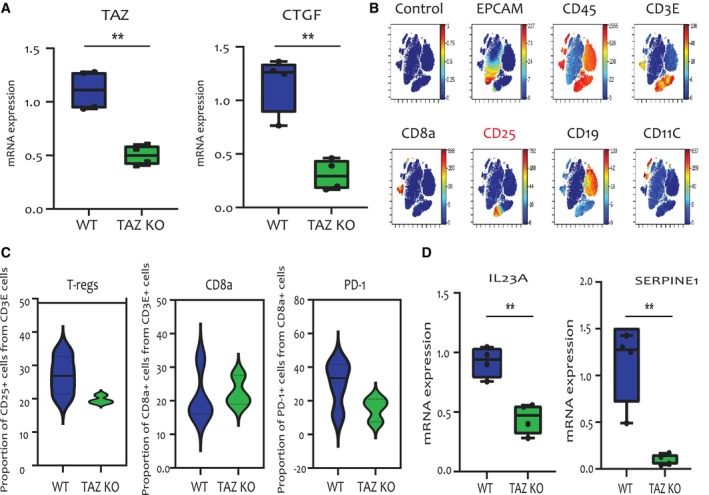
Cytometry by time of flight (CyTOF) analysis of TAZ‐deficient vs. TAZ‐proficient tumours. (A) Relative levels of *TAZ* and *CTGF* mRNA in EPCAM‐positive cells isolated from wild‐type (WT) tumours (*n* = 4) and TAZ‐knockout (KO) tumours (*n* = 3), measured by RT‐qPCR. Error bars indicate mean ± SEM from all repeats. **P < 0.01, Unpaired *T*‐test. (B) Clustering analysis of immune cell populations performed on CyTOF data from a representative sample. Unique cell populations are identified by the markers shown above each graph. (C) Relative proportion of Tregs, CD8^+^ T cells and PD‐1‐positive CD8^+^ T cells in WT (*n* = 4) and TAZ KO tumours (*n* = 3), analysed using Gating Editor in Cytobank [74]. (D) Relative levels of *IL23A* and *SERPINE1* mRNA in the same EPCAM‐positive cells as in (A), determined by RT‐qPCR. Error bars indicate mean ± SEM from all repeats. **P < 0.01, Unpaired *T*‐test. *CTGF*, Connective tissue growth factor; *Il23*, Interleukin 23; *SerpinE1*, Plasminogen activator inhibitor 1.

To further validate the impact of TAZ on the immune TME, we performed mass cytometry analysis by Time of Flight (CyTOF) on TAZ‐proficient vs. TAZ‐deficient 4T1 tumours. As expected, RT‐qPCR analysis of the EPCAM^+^ cells removed from the tumour cell suspension prior to CyTOF analysis confirmed that expression of both *TAZ* mRNA and the TAZ target *CTGF* was strongly reduced in the TAZ‐KO cancer cells, relative to the TAZ‐WT cancer cells (Fig. 5A).

CyTOF analysis of the EPCAM‐depleted cell population enabled clustering of immune cell subpopulations, based on cell identity markers (Fig. 5B). Notably, this confirmed the higher abundance of Tregs in WT tumours compared to TAZ‐KO tumours (Fig. 5C). Moreover, while no differences in the overall relative abundance of CD8^+^ cells were observed between the two genotypes, the CD8^+^ T cells of the WT tumours displayed a markedly higher relative proportion of PD‐1‐positive cells than those of the TAZ‐KO tumours (Fig. 5C), consistent with the conclusion that TAZ in the cancer cells promotes CD8^+^ T cell exhaustion in the immune TME.

Additionally, RT‐qPCR analysis of RNA extracted from the EPCAM^+^ cell subpopulation (see Fig. 5A), comprising mainly the cancer cells, revealed higher levels of *IL23* and *SERPINE1* mRNA in the WT tumours as compared to the TAZ‐KO tumours (Fig. 5D). This indicates that expression of these cytokines in the cancer cells, shown to be positively regulated by TAZ *in vitro* (Fig. 4), is also similarly regulated within the *in vivo* context of the tumour.

Altogether, these observations further confirm the correlation between TAZ, increased expression of specific cytokines and increased abundance of Tregs in the TME.

### 3.5 TAZ tumour promoting activity is compromised in immune‐deficient mice

**Fig. 6:**
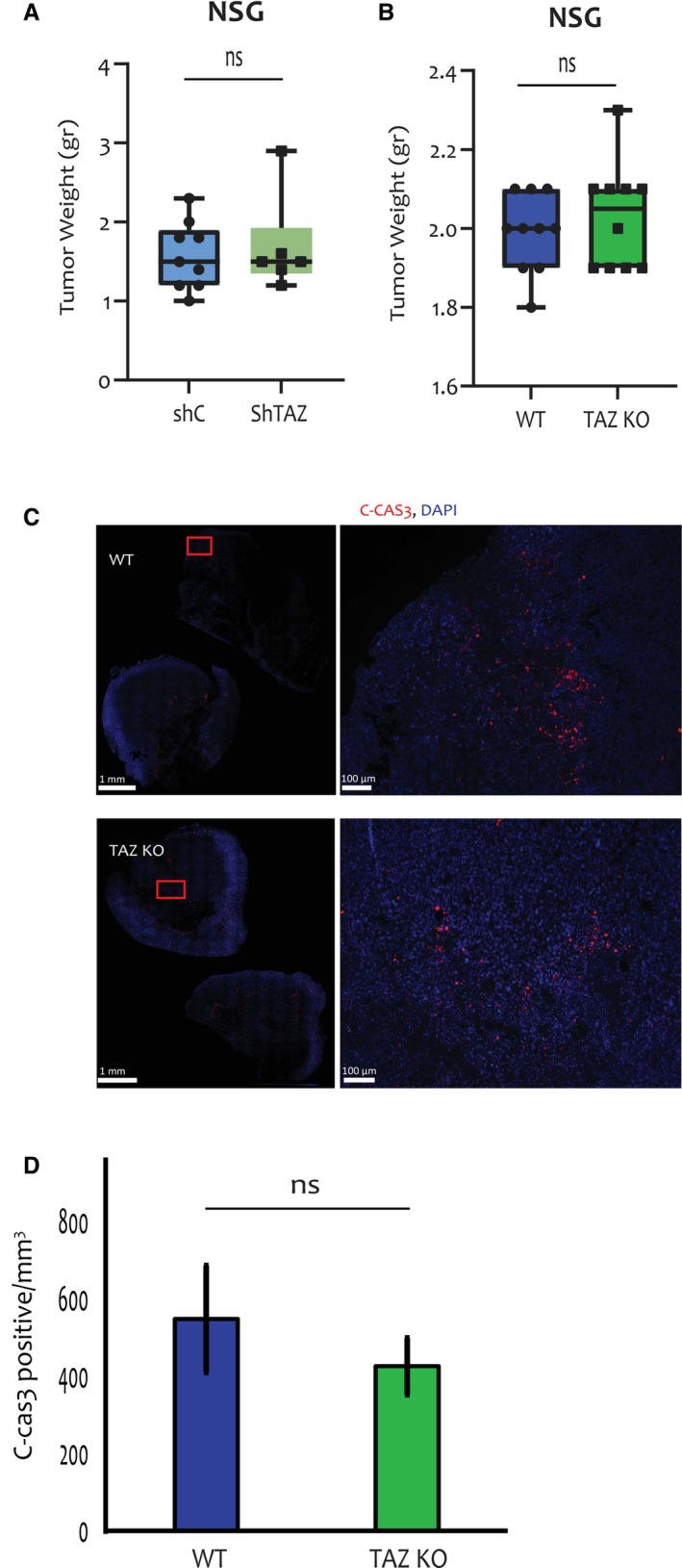
TAZ does not promote tumour growth in immune‐deficient mice. (A) 4T1 cells with inducible shC or shTAZ were treated with 6 μm doxycycline for 48 h prior to orthotopic injection into NSG mice (5 mice per group), pre‐fed with doxycycline chow (625 mg·kg^−1^). Doxycycline chow feeding continued for 5 weeks, when tumours were excised and weighed. Error bars indicate mean ± SEM from all repeats. Unpaired *T*‐test. (B) WT and TAZ‐KO 4T1 cells were orthotopically injected into NSG mice (6 mice per group). Tumours were excised after 5 weeks and weighed. Error bars indicate mean ± SEM from all repeats. Unpaired *T*‐test. (C) Representative images at 1× and10× magnification of C‐Cas3 (red) and DAPI (blue) staining of WT and TAZ‐KO tumour samples from (B). Scale bar = 1 mm or 100 μm for magnified pictures. Magnified sections are marked by red rectangles. (D) Quantification of C‐Cas3 staining from whole slides of WT and TAZ‐KO tumours (6 samples each) excised from NSG mice. Average numbers of C‐Cas3‐positive cells were determined as in Fig. 2G. Error bars indicate mean ± SEM from all repeats. Unpaired *T*‐test. C‐Cas3, cleaved caspase 3; KO, knockout; WT, wild type.

The observed effects of TAZ on T cells and cytokine secretion suggested that its tumour promoting activity might be due, at least in part, to its ability to modulate anti‐tumour immunity. To explore this suggestion more directly, we compared the tumorigenic capacities of TAZ‐depleted vs. TAZ‐proficient cells in 2 immune‐deficient mouse models: nude mice, which have greatly reduced numbers of T cells, and NSG mice, which lack also B cells and NK cells. Strikingly, in both knockdown and knockout models, injection of the cancer cells into immune‐deficient mice (Fig. 6A,B and Fig. S4a,b) abolished the TAZ‐dependent differences in tumour size. This was particularly remarkable for the TAZ‐KO cells, which completely failed to elicit tumours in immune‐competent mice (Fig. 1C), but were as tumorigenic as WT control cells in NSG mice. In accordance, we observed no differences in C‐Cas3 staining between TAZ proficient (WT, shC) and TAZ deficient (KO, ShTAZ) tumours from NSG mice (Fig. 6C,D and Fig. S4c,d), suggesting that the differences in tumour cell death were dependent on immune activity. Together, our observations suggest that the oncogenic activity of TAZ in this setting, and presumably also in human breast cancer, relies at least in part on its ability to modulate the immune TME and suppress anti‐tumour immunity, thereby augmenting cancer cell survival and favouring tumour progression.

## 4 Discussion

TNBC is a highly aggressive subtype of breast cancer [42]. Human breast cancers often thrive by eliciting the overexpression of oncogenes. Thus, it is not surprising that the TAZ oncogene [13, 16, 43, 44] is preferentially overexpressed in TNBCs [45].

The oncogenic activity of TAZ has been attributed to a variety of cancer cell‐intrinsic activities, such as induction of stem cell features and enhancement of migration, survival and anchorage‐independent growth [15, 46, 47]. We now report that TAZ can also regulate the expression and secretion of a number of cytokines, which are capable of modulating the immune tumour microenvironment (TME) and in particular are known to elicit an inflamed, immunosuppressive TME [48, 49, 50, 51, 52, 53].

One of the cytokines secreted by the cancer cells in a TAZ‐dependent manner is IL‐23. IL‐23 can promote the expansion of Th17 cells, thereby altering the immune TME [54]. Interestingly, engagement of the IL‐23 receptor in Tregs activates STAT3, leading to the transcription of FOXP3 and IL‐10 [55]. In this manner, TAZ‐dependent IL‐23 expression may increase the abundance and functionality of Tregs in the immune TME. An additional TAZ‐induced cytokine is Wisp1, which has been implicated in regulating the infiltration of several immune cell types [48]. In invasive breast cancer, Wisp1 has been suggested to inhibit type 1 cell‐mediated immunity by blocking IL‐12 signaling and promoting type 2 immunity [56]. Likewise, Serpin E1 has also been shown to modulate the immune TME, as described for colon cancer [50]. Together, these cytokines are believed to play significant roles in tumour progression by exerting a wide range of effects on the immune TME.

Furthermore, in line with our observations, expression of these cytokines is associated with poor survival [48, 49, 50, 51, 52, 53]. Concordantly, we show that tumours harbouring endogenous TAZ exhibit increased Treg infiltration, which presumably dampens CD8 T cell cytotoxic activity, leading to increased cancer cell viability and overall more robust tumour growth. Importantly, we show that immune cell composition patterns associated with TAZ expression levels in 4T1 mouse tumours are recapitulated in human TNBC breast cancer patients.

Of note, TAZ binds SMAD family members, such as heteromeric SMAD2/3‐4 complexes and SMAD7, and regulates their nuclear localization, transcriptional output and phenotypic impact [57, 58, 59]. The effects of TAZ on TNBC tumour progression, including also modulation of the immune TME, might therefore be modulated in part via interactions not only with TEADs but also with other transcription factors such as SMAD2/3 or SMAD7, which regulates Treg migration into tumours [60].

Notably, deregulation of the Hippo pathway in cancer exerts a broad spectrum of effects, depending on tumour type and tissue context [61, 62]. This may also entail other immunomodulatory mechanisms, in addition to those described above. Thus, YAP and TAZ have been reported to increase the expression of PD‐L1, which can promote tumour evasion by directly inactivating cytotoxic T cells [21, 63, 64, 65]. Furthermore, YAP and TAZ can affect interferon gamma (IFNγ) expression, thereby also modulating inflammatory responses [66].

While this manuscript was in preparation for submission, another study was published that similarly implicated TAZ in mobilizing an immune suppressive TME [67]. In that study, TAZ‐dependent tumour growth was ascribed primarily to the increased recruitment of myeloid‐derived suppressor cells (MDSCs), although an increase in Tregs was not observed [67]. Together with our findings, this adds to the growing evidence linking the tumour promoting functions of TAZ to remodelling of the TME. Supporting this notion, TAZ was shown to spur liver inflammation and hepatic cancer by inducing the transcription of inflammatory cytokines and promoting myeloid cell infiltration [18].

In addition to Inflammatory response, other pathways significantly altered following depletion of TAZ *in vivo* involved metabolic functions, specifically fatty acid metabolism (Fig. 3A). Fatty acid metabolism, which is essential for energy production and storage and for cell membrane expansion during cell proliferation, has received increasing attention for its pivotal role in cancer [68]. Interestingly, release of free fatty acids by breast cancer cells has been shown to block the antitumour activity of cytotoxic T cells [69]. Furthermore, TAZ also possesses anti‐apoptotic functions [70], which are expected to dampen the immune‐mediated killing of cancer cells. Hence, TAZ regulates both cell intrinsic (reduced vulnerability to apoptosis and metabolic alterations) and cell non‐intrinsic (cytokine secretion) functions that, together, increase the resilience of the cancer cells in the face of immune surveillance.

YAP, the TAZ paralog, has also been implicated in similarly modulating the immune TME, by recruitment of immunosuppressive macrophages [71] and MDSCs [72, 73]. Thus, both YAP and TAZ, the key Hippo effectors, exert their oncogenic effect largely by reshaping the immune milieu from tumour restrictive to tumour permissive.

In summary, we propose that breast cancer cells maintain TAZ expression to evade immune attack. Blocking TAZ activity within tumour cells might thus have a dual beneficial role, by both suppressing intrinsic malignant features of the cancer cells and reversing the immune suppressive TME, thereby potentially also rendering the tumours more vulnerable to immunotherapy.

## 5 Conclusions

We found that depletion of TAZ in mouse triple‐negative breast cancer cells reduces the ability of such cells to form tumours when injected orthotopically into immunocompetent syngeneic mice. We show that this is due to an ability of TAZ to upregulate the expression and secretion of proteins that orchestrate an immunosuppressive tumour microenvironment, thereby protecting the growing tumour from attack by the adaptive immune system. Importantly, a similar mechanism appears to be operating also in human triple‐negative breast tumours. The existence of such non‐cell autonomous function of TAZ suggests that interference with this function may increase the ability of the immune system to restrain the progression of triple‐negative breast cancer, and perhaps sensitize it to immune checkpoint therapy.

## Conflict of interest

The authors declare no conflict of interest.

## Author contributions

AG, OH, YY and MO conceived and designed the project; AG and OH performed research and acquired data; NBN, SB, AA‐S, ACP, TMS, SM helped with the experiments; LF‐A, TMS, GM helped with the analyses; YY, YA and MO supervised research; AG, OH, YA and MO wrote the paper. All authors discussed the results and commented on the manuscript.

### Peer review

The peer review history for this article is available at https://www.webofscience.com/api/gateway/wos/peer‐review/10.1002/1878‐0261.13525.

## Supporting information


**Fig. S1.** Validation of TAZ‐deficient cells.
**Fig. S2.** TAZ is associated with an increased inflammatory response gene signature.
**Fig. S3.** TAZ promotes cytokine expression and secretion.
**Fig. S4.** TAZ‐dependent differences in tumor size are abolished in immune‐deficient mice.

**Table S1.** Sequences of ShTAZ and scrambled ShC.

**Table S2.** Sequences of primers used in the manuscript.

**Table S3.** Mice used in the experiments.

**Table S4.** Gene lists from the gene set enrichment analysis (GSEA).

## Data Availability

All data needed to evaluate the conclusions in the paper is included in the paper and/or the Supporting Information. Additional data related to this paper is available from the corresponding authors upon request. The data that support the findings of this study are openly available in NCBI's Gene Expression Omnibus and are accessible through https://www.ncbi.nlm.nih.gov/geo/query/acc.cgi?acc=GSE242178, GEO Series accession number GSE242178.
