# Supplementary material for: TAZ facilitates breast tumor growth by promoting an immune‐suppressive tumor microenvironment
Source: Mol Oncol. 2023 Oct 16;17(12):2675–93. doi: 10.1002/1878-0261.13525 (PMC10701768; doi:10.1002/1878-0261.13525)
Supplement: Supplementary file 1 — Fig. S1. Validation of TAZ‐deficient cells. Fig. S2. TAZ is associated with an increased inflammatory response gene signature. Fig. S3. TAZ promotes cytokine expression and secretion. Fig. S4. TAZ‐dependent differences in tumor size are abolished in immune‐deficient mice. [file MOL2-17-2675-s003.zip › Supplementary Figure legends.docx]

**Supplementary Figure legends**

Supplementary Figure S1: Validation of TAZ-deficient cells

(a) Sequence of the *Taz* sgRNA and localization of its target within the mouse *Taz* (*Wwtr1*) gene. Rectangles mark axons, lines mark introns. (b) DECODR output table depicting sequencing data of two independent *Taz* KO clones. Left to right: name of clone, calculated numbers of all possible indels, estimated percentage of presence in the tetraploid 4T1 genome, and sequencing data aligned to the WT sequence. (c) Relative *Taz* mRNA levels in WT clones (averaged from 3 clones) and TAZ-KO clones (averaged from 2 clones), determined by RT-qPCR 48 hours after cell seeding. 3 technical repeats for each clone. (d) Relative *Taz* mRNA levels in control (shC) cells and shTAZ cells, determined after 48 hours of treatment with 6µM doxycycline. Data from 3 biological repeats. p-value= 0.0004. (e) Percentage of Propidium iodide (PI) positive shC vs shTAZ cells after 48 hours of treatment with 6µM doxycycline. PI staining was analyzed using a Guava benchtop FACS machine. Average of 4 biological repeats. n.s. = not significant. (f) Western blot analysis of TAZ, YAP and GAPDH proteins in representative WT and TAZ-KO 4T1 single cell clones. SgRNA: single guide RNA, DECODR: DEconvolution of COmplex DNA Repair, WT: Wild type, KO: Knockout. YAP: Yes associated protein.

Supplementary Figure S2: TAZ is associated with an increased inflammatory response gene signature

(a) Gene Set Enrichment Analysis (GSEA) of the Inflammatory response gene signature (derived from the comparison of shC vs shTAZ tumors, see Supplementary table S4) vs the *in vitro* gene expression data of shC compared to shTAZ cells, treated for 48 hours with 6µM doxycycline. (b) GSEA of the Inflammatory response gene signature in (a) vs the *in vitro* gene expression data of WT cells compared to TAZ-KO cells. (c) GSEA of the YAP/TAZ target genes signature^80^ in HTAZq vs LTAZq human TNBC tumors, defined as in Fig. 4c. (d) GSEA of the Inflammatory response gene signature as in (a) in HTAZq vs LTAZq human TNBC tumors. (e) CIBERSORT plot of the relative abundance of different types of immune cells (fraction out of the total) in shC vs. shTAZ mouse tumors (top) and in HTAZq vs. LTAZq human TNBC tumors (bottom). Tregs are highlighted. (f) Relative expression of a Treg signature^81^ in HTAZq vs LTAZq human TNBC tumors. p-value = 0.023. (g) Scatter plot of the correlation between the Treg gene signature in (f) and the Inflammatory response gene signature (derived from the comparison of shC vs shTAZ mouse tumors and converted to the human counterparts) in TCGA breast cancer tumor samples. (h) Quantification of CD3-positive cells in shC vs shTAZ tumors (4 tumors each). 6 representative fields from each tumor were analyzed using ImageJ. (i) Quantification of the fraction of FOXP3 positive cells, out of the total number of CD3 positive cells, in shC vs shTAZ tumors (4 tumors each). 6 representative fields from each tumor were analyzed using ImageJ. (j) Representative fields of WT, TAZ KO, shC and shTAZ tumor samples, stained for DAPI (blue) CD3 (red) and FOXP3 (green). Images were taken at 10x magnification. YAP: Yes associated protein, WT: Wild type, KO: Knockout, HTAZq: Highest quartile *TAZ* mRNA expression, LTAZq: Lowest quartile *TAZ* mRNA expression, FOXP3: forkhead box P3. TCGA: The Cancer Genome Atlas.

Supplementary Figure S3: TAZ promotes cytokine expression and secretion

(a) Images of the cytokine array blots from WT and TAZ-KO cells. Analysis was performed using ImageJ. Black rectangle = positive control reference dots, red rectangle = WISP-1 dots as an example of a significantly different cytokine (see Fig. 4a). (b) Representative image of the cytokine array blots from shC and shTAZ cells, marked as in (a). (c) RT-qPCR analysis of the indicated cytokine mRNAs in shC vs shTAZ cells treated for 48 hours with 6µM doxycycline. Data from 3 biological repeats. Values are normalized to shC. p-values, from left to right: 0.005, 0.008, 0.19. (d) Binding of TAZ and TEAD4 to regulatory regions of the human *IL23a* gene, plotted as described in Fig. 4d. (e) Correlation between expression of the Treg signature (Fig. S2f) and the cytokine array signature (*Serpine1, CCN4* and *IL23a*) in TCGA breast cancer tumor samples. Tumors are colored according to their relative *TAZ* mRNA expression (red = high expression, blue = low expression). (f) Kaplan-Meier relapse free survival (RFS) plot of all breast cancer patients, comparing low expression (black) vs. high expression (red) of TAZ (202133_at). High and low expression were defined as being above or below the median, respectively. WT: Wild type, KO: Knock out, ShTAZ: *TAZ*-targeting shRNA, ShC: scrambled shRNA. *SerpinE1*: Plasminogen activator inhibitor 1, *WISP1/CCN4*: CCN family member 4, *Il23*: Interleukin 23.

Supplementary Figure S4: TAZ-dependent differences in tumor size are abolished in immune-deficient mice

(a) WT and TAZ-KO 4T1 cells were orthotopically injected into Nude mice (n=3 for WT and n=5 for TAZ KO). Tumors were excised after 5 weeks and weighed. (b) 4T1 cells with inducible shC or shTAZ were treated with 6µM doxycycline for 48 hours prior to orthotopic injection into Nude mice (12 mice in shC group and 8 mice in shTAZ group), pre-fed with doxycycline chow (625mg/kg). Doxycycline chow feeding continued for 5 weeks, when tumors were excised and weighed. (c) Representative images at 10x magnification of C-Cas3 (red) and DAPI (blue) staining of shC and shTAZ tumor samples from (b). Scale bar= 100µm. (d) Quantification of C-Cas3 staining from whole slides of shC and shTAZ tumors (5 samples each) excised from Nude mice. Average numbers of C-Cas3-positive cells were determined as in Fig. 2g. WT: Wild type, KO: Knockout, ShTAZ: *TAZ*-targeting shRNA, ShC: scrambled shRNA, C-Cas3: cleaved caspase 3.
